# Veterinary ethics and companion animal euthanasia: what can we learn from critical disability studies?

**DOI:** 10.3389/fvets.2024.1412327

**Published:** 2024-10-25

**Authors:** Jamie Arathoon, Lauren Van Patter

**Affiliations:** ^1^School of Geographical and Earth Sciences, University of Glasgow, Glasgow, Scotland, United Kingdom; ^2^Department of Clinical Studies, Ontario Veterinary College, University of Guelph, Guelph, ON, Canada

**Keywords:** animal studies, critical disability studies, end-of-life, euthanasia, veterinary ethics, veterinary humanities, veterinary medicine

## Abstract

Within veterinary ethics and practice around companion animal end-of-life and euthanasia, the political and cultural dimensions of death and dying are rarely addressed. This reduces the ability to engage with questions like: what future potential goods (positive affective states, meaning) could an animal experience by continuing to live; what constitutes a life worth living; and how can we make this decision for another being? These are questions that have been subject to extensive dialogue within Critical Disability Studies. The aim of this paper is to provide an analysis of how core considerations from Critical Disability Studies could be useful in veterinary ethics when considering companion animal end of life and euthanasia. First, critiques of the *dis/ability binary* and associated hierarchies raise questions about how animal disability and illness are understood, and around challenging questions like psychological illness and behavioural euthanasia. Second, nuanced engagements with questions of *a life worth living* and Quality of Life emphasise the importance of individual animal experiences, personality, and the foregrounding of ‘what is important for the animal’. Third, dialogues around *choice and agency* critique the tendency to focus on owner choice, rather asking what opportunities there are to listen to the preferences of animals themselves. Finally, engaging with *care and power* highlights the ambivalent nature of caregiving, of euthanasia as a practice of care, and the power intrinsic to making End of Life decisions on behalf of another. Overall, perspectives from Critical Disability Studies allow us to engage with challenging questions of veterinary ethics and end-of-life care in companion animal practice with more nuance and complexity.

## 1 Introduction

End-of-life (EoL) decision-making and euthanasia are some of the most challenging areas within veterinary medical practice and ethics. Euthanasia and EoL care are challenging because some evaluation scales exist for some chronic illness and degenerative conditions, and not others, and in both cases, whether a scale is present or not, this requires owners and veterinarians to decide what is best. Further challenges include different veterinary and owner understandings of suffering and wellbeing. Although most veterinary schools teach about euthanasia and other EoL topics (e.g., analgesia, communication with owners^1^), often there is very little time spent on the ethical and cultural dimensions of death and dying (1). Veterinary ethics tends to be taught from a utilitarian standpoint, where the emphasis is on the prevention of animal suffering (2). If euthanasia constitutes a painless death, it thus becomes positioned as unproblematic, and in many cases is argued based on the prevention of future animal suffering. What is often absent from the moral calculus are questions like: what future potential goods (positive affective states, meaning) could the animal experience by continuing to live? What constitutes a life worth living, and how can we make this decision for another being?

These are questions that have been subject to extensive dialogue within Critical Disability Studies (CDS). CDS is a branch of Disability Studies that brings in intersectional perspectives from postcolonial, posthumanist, feminist, and Queer theories to trouble ideas about disability and normality (3–7). CDS maintains a commitment to politically organise and contest dis/ablism in everyday life, while being mindful of different socio-cultural, historic, and economic contexts (4). CDS has generally focused on humans exclusively, but recently there has been an increase in engagement with Animal Studies in topics as varying as the intersection of disability and animal justice and the disabling of industrially farmed animals for food production (8–12). For instance, S. Taylor (12) writes about how breeding farmed animals for increased production efficiency has resulted in many disabling conditions, such as chickens and turkeys whose legs cannot bear the weight of their breasts. As another example, Guenther (13) considers how animal rescuers and shelter volunteers both advocate for dogs with disabilities, while reinforcing rhetoric that perpetuates disability as tragic, a dis/ability binary, and logics of saviourism. To date, there has been very limited dialogue between veterinary ethics and CDS, but we feel CDS has a great deal to offer in terms of understanding the complexity of dimensions like what constitutes a life worth living which inform ethical concerns around EoL and euthanasia in veterinary medicine.

Our aim of this paper is to provide an analysis of how core considerations from CDS could be useful in veterinary ethics when considering companion animal end of life and euthanasia. We focus on companion animal practice, as this constitutes the bulk of the literature on veterinary ethics, and the discourses and practices within other contexts such as animals enrolled in agricultural uses, or wildlife, look very different. We are critical/cultural geographers writing from the context of the United Kingdom and North America, and much of the discussion focuses on work in these contexts. One of us has focused on questions of care in assistance dog partnerships, and of social justice across animal and disability geographies, and has lived experience of depression and anxiety that continually informs their approach to these topics. One of us has focused on questions of ‘living well’ in multispecies communities and equity and justice in access to animal healthcare and has lived experience of disability (Traumatic Brain Injury) that shapes their thinking and praxis. Below we briefly summarise the state of the discourse and literature on euthanasia in companion animal veterinary practice, followed by CDS and how it has intersected with Animal Studies. We then present four areas in which we feel veterinary ethics could be informed by dialogues in CDS: the dis/ability binary; a life worth living; choice and agency; and care and power. In each section we provide an overview of key concepts and conversations in CDS, followed by a consideration of its relevance to veterinary ethics, euthanasia, and EoL conversations in the context of companion animals.

## 2 Euthanasia and ethics in companion animal veterinary practice

The Canadian Veterinary Medical Association (14) defines euthanasia as “the act of intentionally and humanely ending the life of an animal.” It is often framed around offering a ‘good death’, meaning one in which suffering is minimised. The British Veterinary Association [(15), emphasis in original] has three categories of euthanasia:

*(1) **Absolutely justified** euthanasia: the only option based on the welfare of the animal; (2) **Contextually justified** euthanasia: treatment is available but may not be the best option in the circumstances (eg, unpredictable aggression particularly towards children); (3) **Non-justified** euthanasia: a variety of alternatives exist, such as rehoming, but are refused by the owner.*

This is suggestive of the moral dimensions of euthanasia—that as an EoL practice or process, it can be more or less justifiable.

The American Veterinary Medical Association (AVMA) [(16), p. 6] guidelines on euthanasia specify that euthanasia constitutes a “good death” when “death is a welcome event” and “continued existence is not an attractive option.” The argument is that when suffering is sufficiently great, the animal no longer has an interest in continuing to live, or continuing to live is a worse outcome than death. Utilitarian logistics are often central to such decision making: euthanasia prevents future harms, and if the animal is in a state of suffering that is unlikely to abate, then one is not depriving them of future goods since little opportunity for experiencing goods such as pleasure or joy remain when one is in a state of suffering. There is no single accepted definition of suffering within veterinary literature and practice, and it can be challenging to define and assess suffering (16). Discussions of suffering and quality of life frequently consider factors including physical pain and psychological wellbeing from both the veterinarian and owner’s perspectives, including: clinical signs of disease progression; behavioural indicators of pain; the individual’s ability to perform essential tasks such as eating and drinking, sleeping, grooming; their ability to engage in activities they enjoy; and how many ‘good’ versus ‘bad’ days the individual is having (16–19).

The status of other-than-human animals as property rather than persons—that is, as entities that do not qualify as rights bearers under modern liberal/settler-colonial socio-legal structures—also influences euthanasia and EoL decision making. For instance, the AVMA Guidelines [(20), p. 8] specify that decisions around euthanasia may be “complicated by external factors, such as productivity, the greater public and general good, economics,” for instance in the case of animals used for research. Conversely to humans, who are in principle and by law generally owed the same fundamental rights in all circumstances (though of course one could object to how this plays out for those who are incarcerated, prisoners of war) the experience of animals, including those who are euthanized, vary greatly by species and location. Even cats and dogs who are generally afforded the greatest protection and consideration, face very different opportunities depending on whether they are classified as ‘pets’, ‘laboratory animals’, or ‘pests’. For instance, Johnston (21), details the example of unowned cats in Miami, who are only legally permitted to be euthanized through barbiturate injection within shelters, but can legally be killed by gassing if trapped as nuisance wildlife on the streets. The AVMA Guidelines (20) elaborate that:

*Euthanasia protocols for companion animals (usually dogs and cats) in institutional settings (eg, shelters, large breeding facilities, research facilities, quarantine facilities, racetracks) may differ from those applied in traditional companion animal clinical practices due to situation-specific requirements, including variable access to pharmaceuticals and other equipment, diagnostic and research needs (eg, postmortem tissue samples), and the number of animals to be euthanized* (p. 56).

These considerations are very different from those that would be found around EoL in the context of human medicine. While EoL decisions for humans are informed by legal contexts (e.g., availability of medically assisted dying), there are more often additional dynamics under consideration, such as palliative care options including sedation, pain management, and spiritual counselling (22).

Veterinary ethics is a relatively nascent subfield that has been growing as the ethical quandaries central to the profession receive increasing attention. Woods [(23), p. 13] writes that over the last decades, “veterinarians began to recognize the potential conflicts in interest between the animal, owner, society and profession.” As noted above, veterinary ethics has been largely grounded in utilitarian (consequentialist) philosophical traditions. Utilitarianism involves moral decision making that seeks to maximise benefits and minimise harms, weighing the various anticipated outcomes of different decisions. Feminist ethics of care has push back against assumptions that we can make moral decisions objectively from a place of detachment, reducing ethics to a simple calculus. Rather, they embrace a contextual, relationship-centred approach to ethical decision making which foregrounds the importance of emotions, vulnerability, and responsibility (24–26). Recently, Ashall has argued for a ‘feminist veterinary ethics’, noting that “traditional reductionist philosophy … has embedded within veterinary ethics the language of rules, calculations and impartiality” [(2), p. 11]. Conversely, a feminist ethics of care can augment the focus on the “previously under acknowledged emotional, relational and contextual realities of veterinary practice” (p. 11), including EoL decision making and practices.

## 3 Critical disability studies and the animal

Disability Studies was first developed in the 1980s by Mike Oliver through his seminal book *Social Work with Disabled People* (27). Oliver introduced the social model of disability, which, rather than understanding disability as a medical ailment that impairs people who need a cure, focuses on socio-political conditions which give rise to disabling environments and societal responsibilities for accessibility and equity for those living with different capabilities. This work, among others (28–30), challenges the dominant medical model of disability which defines disability as a “pathological condition, as deficit, and, significantly, as an individual burden and personal tragedy” [(31), p. 11]. Since the 1980s there have been several different theoretical models championed by Disability Studies scholars in an attempt to understand the lived experience of disability (32–34). CDS as a branch of disability studies developed later with some of the key social justice goals of disability studies in mind, but to bring in intersectional perspectives to trouble ideas about disability and normality in challenge to deconstruct the binaries of able/disabled, healthy/ill, sane/insane (3–7).

In the social sciences and humanities there has been growing attention paid to the shared challenges that both persons with disabilities and nonhuman animals face (8–12, 35). Critical Animal Studies (CAS) is interested in ethical praxis wherein animals are not exploited for capitalist gain. As N. Taylor and Twine [(36), p. 1] state “CAS seeks to differentiate itself from the broader AS [animal studies] field by having a direct focus on the circumstances and treatment of animals.” CAS is influenced by anarchist, feminist, liberationist, and anti-capitalist thought and practice. It is grounded in beliefs that research should be orientated around political change for animals from the oppressive systems that exert power over them.

At the intersection of CAS and CDS are challenges and spaces for opportunity. Despite healthy debate CAS and CDS have often been cast at odds with one another. Animality has been used to dehumanise persons with disabilities while ableist logics, particularly around cognition, have been used to exclude animals from moral consideration (8). One clear example is the work of philosopher Peter Singer (37, 38), who enrolled problematic stereotypes and discourses around persons with disabilities and animals. Singer’s utilitarian arguments, based on notions of rationality, consciousness, and autonomy, have been used to devalue persons with disabilities in an attempt to argue for the greater rights of animals. Singer has come under heavy critique from CDS scholars for his use of stereotypes, assumptions on suffering, and understanding of rationality, that perpetuate eugenic logics towards persons with disabilities (6, 8, 10, 12). These utilitarian ideals have positioned animals as in need of equal forms of care to humans based on the idea that persons with disabilities are unable to express desires beyond biological needs and lack rational thought, whereas some animals can show rational thought.

Despite these tensions, both CAS and CDS seek pathways to social justice by challenging power and oppression, in attempting to understand lived experiences. As Jampel [(39), p. 125] argues, disability justice is collective justice, it “includes a commitment to addressing multiple forms of oppression.” This is tied to S. Taylor’s [(12), p. 146] statement that “we cannot have disability liberation without animal liberation—they are intimately tied together.” One way to approach this is through analysing “spaces of speciesist and disabling violence” [(8), p. 4], which arguably intersect within medical practices around disability and EoL for both humans and other animals.

Within the human medical context, there is an understanding that “many people with disabilities tend to distrust how medical professionals (de)value their lives and (mis)interpret their quality of life” [(40), p. 115]. Despite much work and activism in CDS, “the political context of disability studies has not penetrated either clinical ethics or student training” [(40), p. 115]. We argue that similarly, veterinary medicine’s approach to disability, illness, and aging in animal patients still relies on a medical model of disability, which informs end of life decision-making.

## 4 Bringing CDS into conversation with veterinary euthanasia: four considerations

### 4.1 The dis/ability binary

Disability and ability have often been constructed as binary concepts where disability implies a lack of ability to do specific things (e.g., walking up steps). Representations of disability profoundly shape what society thinks specific bodies can or cannot do. In this sense disability has often been denigrated throughout history, constructed as less-than, with persons with disabilities marginalised by, and excluded from, spaces designed by and for people who have perceived ‘normal’ abilities. In dominant discourses, disability is reproduced as oppositional to normative, non-disabled society (41).

CDS scholars have attempted to unpack several issues with the dis/ability binary, including that it leaves little space for the notion of in-betweenness. Binaries of disabled/able and healthy/ill, have systemically resulted in an organisation of society in which bodies cannot occupy, or act, as *both* healthy/ill and able/disabled (42). Despite these understandings, many disabilities occupy both categories due to their episodic nature. For instance, chronic pain and illness are about being both healthy and ill at the same time. Dominant understandings of dis/ability leave no space for this uncertainty, indeterminacy, or fluctuation.

CDS scholars destabilise the dis/ability binary through considering dis/ability as a split term. As Goodley et al. (4) state:

*Dis/ability is a split term – and a term split for a reason – to consider the ways in which disability/ability are always reliant upon one another (an obvious point), and in order to think of disability we must pull into the foreground the entity that is ability (a less well developed idea). To know something about disability one needs to have a sense of its often hidden referent (ability)* (p. 986).

These binaries can be subverted by understanding that rather than some of us being autonomous, independent individuals with few limitations (which is lauded) and others being dependent and with limitations (which is denigrated), we are all vulnerable and interdependent (12) in various ways, and at varying times in our lives. Further destabilisation can occur by “shifting the emphasis from (aiding disabled people in) *doing things ‘normally’* to (underlining for all of ‘us’) simply the *normality of doing things differently*” [(43), *emphasis original*, p. 493]. Instead of structuring the world along the lines of normative/*deviant*, we can understand the world, our abilities, and experiences as infinitely *diverse*, we can move away from problematic narratives grounded in binaries and hierarchies.

#### 4.1.1 Relevance to veterinary ethics and euthanasia

In decisions around animals’ quality of life (QoL) and euthanasia, whether the animal is healthy *or* ill, well *or* unwell, is often central to the conversation. But similar to humans, how do we conceptualise and make decisions when wellness is not fixed, but fluctuating? When an individual may be less able than what is considered as normative along some axes, but still able to do many things - is it similarly complicated and problematic to attempt to classify animals as ‘healthy’ or ‘normal’ versus not? Understandings of disability get problematically projected onto other animals, although disability itself is a social construction that may or may not have any meaning for other species. Countering the narratives that “the natural process for a disabled animal is to die” (p. 26–27), S. Taylor (12) writes that “examples of disability survival, adaptation, and care in the animal world” (p. 28) are prominent in many species, from elephants, apes, dogs, pigs, and turkeys. S. Taylor invites us to think about how ‘animal crips’ challenge “us to question our ideas about how bodies move, think, and feel and what makes a body valuable, exploitable, useful, or disposable” (p. 43).

An interesting question related to veterinary ethics and understandings of disability is behavioural euthanasia—where companion animals are euthanized for perceived behavioural issues like reactivity or anxiety. Separation anxiety for instance can result in undesirable behaviours such as excessive vocalisation, inappropriate elimination, and destructive behaviours (44). One UK study found that for dogs under the age of three, the most common reason for euthanasia (33.7% of cases) was undesirable behaviours (45). Similar findings have been reported in Australia (46). There are three common justifications for such behavioural euthanasia. The first is public safety, when aggression presents risks of dog bites. The second is owners not having sufficient resources or the ability to undertake the rehabilitation required to curb the behaviour. One study reported that short-term (2–8 week) hospitalisation in a shelter equipped to treat such dogs with cognitive/behavioural and pharmacological therapies was an effective alternative to euthanasia (47). The comparison to human psychiatric institutionalisation was made explicit, which raises interesting questions about how non-physical disabilities are conceptualised and approached in companion animals.

The third and most relevant justification is that the measures needed to keep people and other animals safe from dogs exhibiting fear-based aggression, or required to keep the dog from injuring themself (such as prolonged crating), are so restrictive that they potentially make the life of that individual no longer worth living: “Do they ever get to be a dog? In some situations, the measures you need to enact for safety may be extreme and euthanasia could be the kinder option” (48). Likewise, as Heinrich and Clader (49) state:

*Emotional well-being and mental suffering may not be as visible to us as physical pain and disease but can significantly affect your pet’s quality of life and, therefore, yours. When making euthanasia decisions, it is important to consider your pet’s overall emotional state and well-being* (n.p.).

Such sentiments reflect ongoing debates within human medicine around medical assistance in dying (MAiD) and what is termed ‘irremediable psychiatric suffering’ (50, 51).

In companion animals, undesirable behaviours which are perceived as dangerous, or simply inconvenient, are seen as sufficient justification for euthanasia, rendering ‘deviant’ animals killable, reflecting eugenicist histories of psychiatric disorders in the last century (52). These dynamics illustrate that understandings of being healthy/ unhealthy are not such clear binaries, where dogs who physiologically may have an absence of illness and suffering that would typically be used to justify euthanasia may be understood as psychologically unwell or experiencing sufficient ‘mental suffering’ to position euthanasia as a compassionate option.

### 4.2 A life worth living

The concept of a ‘life worth living’ has been critiqued by CDS, as it “has always been a question of disability” (53). Reynolds (53) discusses how this has been conceptualised within western philosophy, concluding that “The canonical idea that some lives are not worth living results from the ableist conflation of disability with pain and suffering.” They write that there has been a lack of attention to the meaning and definition of the concepts ‘disability’, ‘harm’, ‘pain’, and ‘suffering’, as well as to the relationships between each of these. These conceptual questions pertaining to ‘lives worth living’ have material consequences: they impact policies and practices, for instance around euthanasia, selective abortion, genetic testing, loss of life, and extreme poverty (54). They risk eugenic discourses and practices when some lives are deemed as more worthy than others, namely those that are able-bodied/able-minded.

Within medical practice, the prevalent ‘medical model of disability’ has been critiqued for getting “experiences of disability and pain so wrong because of its implicit conception of ability - ability as personal control” (53). In medical practice, this model often “flattens communication about disability to communication about pain, suffering, hardship, undesirable experiences, morbidity, and mortality” (53). In other words, it assumes a particular understanding of disability and its meaning—as a lack, or deficiency—rather than inviting an understanding grounded in an appreciation for difference, and attention to the particularities and nuances of all the many ways one may live within these body-minds. This may lead to practitioners seeing QoL as lacking, even when a patient’s lived experience of disability may not reflect this, leading to “the confounding of disability with end-of-life” [(40), p. 116].

K. Hall [(55), p. 6] writes about a life worth living, arguing that rather than “a question about whether disability impoverishes or enhances quality of life,” it is a question about “what makes possible a life that can be lived.” This points to an understanding of lived experiences of disability as shaped by social factors, including available supports, and ‘caring systems’ (53). As Wiebe and Mullin (56) discuss, poor QoL in some circumstances may have less to do directly with experiences of disability, and more to do with unjust systems which create disabling conditions, such as lack of social and economic support, leading to suffering or despair.

#### 4.2.1 Relevance to veterinary ethics and euthanasia

For animals, ‘a life worth living’ is often written about and assessed using QoL as a measurement^2^ where both veterinarian and owner can subjectively address questions about the animal. The International Association of Animal Hospice and Palliative Care Guidelines (57) state that:

*Quality of life refers to the total well-being of an individual animal, taking into account the physical, social, and emotional components of the animal’s life. Within hospice care, assessments of an animal’s quality of life typically reflect how an animal’s total well-being is affected by disease, disability, or changes related to advanced age* (p. 9).

Dickinson and Hoffmann [(58), p. 57] discuss the difficulty of this measure, finding that while scales of QoL are useful as a more objective marker, there are challenges in answering, “What is the quality of life (QOL) of the animal?” Downing et al. (59) suggests that veterinarians should discuss QoL of the companion animal with owners in terms of what the patient does differently now compared to before the illness. As one example of how QoL is assessed, Littlewood et al. (60), explore how owners of older and chronically ill cats make EoL decisions. Changes in eating habits and weight were seen as primary indicators of QoL decline, as they are more easily observable compared with factors like pain. Owners struggled to evaluate pain, and to distinguish between ‘normal’ aging and poor QoL. Others, such as Lynch et al. (17), when addressing QoL in cats and dogs with cancer, use parameters such as happiness, mental status, pain, appetite, hygiene, water intake, mobility, and general health, within questionnaires. While some of these indicators are generally accepted, there were additions and removals suggested by both veterinarians and owners around both identified symptoms and general understandings of the questionnaire.

Another related concept to QoL is ‘quality-adjusted life years’ (QALY), or how much ‘quality’ time the individual would have left in their life given various intervention options (22). In the case of treatment that causes suffering, anticipated QALY is often weighted against anticipated suffering and duration involved in various interventions. These questions are relevant in both the human and animal medical contexts, however, one often noted difference is that animals (as well as some humans, such as young children) do not have the capacity to understand the potential long-term benefits that could come from short-term suffering, for instance chemotherapy with a good chance of curing cancer that would otherwise be terminal. Thus, often in the case of animals, the suffering of treatment is not considered worth the potential positive prognosis since the animal will be mired in their experience of suffering without any concept of eventual recovery or a return to ‘higher quality’ life post-treatment (22).

The idea of QALY brings to the fore questions of age and aging, something given much consideration in the disability studies community. As one example, the Scandinavian Model of Successful Aging (61) does not focus on individual bodily attributes when it comes to aging and disability, but rather on living conditions and societal responsibilities to enable older individuals with disabilities to have an active engagement with life. Similarly, we might ask what living conditions can be fostered that enable aging companion animals to maintain an active engagement with the things that matter to them, even if this looks different from before. For example, assistive technologies like wheelchairs can allow dogs to continue being mobile, or walking routines can be maintained for dogs using pushchairs. Relatively simple modifications to the environment can be enabling, like elevated feeding stations, ramps onto beds, couches, or window ledges, carpeting or padding over slippery surfaces, and extra padding on areas where cats might jump down from a height.^3^ Understandings of an ‘active engagement with life’ might change over an individuals’ life, and need to be fluid. Individual family circumstances and capabilities also need to be considered. Not every person with a dog with mobility issues has access to or could afford a mobility aid—but the more these modifications and interventions become available and normalised, the more supporting companion animals through aging or disability will seem feasible to families, rather than unreasonable or too taxing.

It is also important to consider that while QoL measures might be appealing in their perceived ability to render qualitative and values-based judgements more objective, culturally-embedded views on pain, suffering, and death will inevitably inform what is seen as acceptable QoL and options like euthanasia, palliative, or hospice care. For example, Hurn and Badman-King (62) write about navigating EoL in a multispecies, multi-faith ashram:

*While veterinary approaches to nonhuman suffering treat all suffering as negative and to be avoided, the community’s understanding of spiritual growth is strikingly different. Suffering, which is an inevitable aspect of living and dying, presents additional opportunity for individuals to know themselves and to know God* (p. 143).

Values and cultural assumptions cannot be taken out of the equation, but rather, “[b]oth veterinarians and clients should consider how their own beliefs, values, and preferences might influence QoL assessments” [(63), p. 46]. In addition to cultural beliefs, financial constraints of care and caregiver burnout syndrome are also used as considerations for euthanasia.

Along with not assuming universal understandings of suffering and death and imposing these on others, it is also important to take into account human standpoints, but also the experiences and preferences of the animal themself. As noted in the IAAHPC guidelines [(57), p. 11], QoL assessments “need to reflect what is important for the animal, not the caregiver or animal hospice providers.” This differs from AVMA Guidelines and common treatment of the topic within the veterinary literature, which tends to emphasise human caregiver assessments and preferences. The IAAHPC Guidelines further detail how individual personality and preferences will alter QoL Assessments for different individuals, even if experiencing the same disease or EoL decline:

…*loss of mobility might negatively impact a dog who loves to play ball and Frisbee more significantly than a dog whose favorite activity is sleeping in a sunny spot under a window. Individual animals also have unique capacities to adapt to change. A disabled animal may continue to enjoy his or her favorite activities if creatively modified to fit the animal’s condition. A disabled animal may also develop “new” favorite activities* [(57), p. 10].

This emphasis on individual experiences, personality, and the foregrounding of ‘what is important for the animal’ in QoL determinations leads to considering choice and agency.

### 4.3 Choice and agency

Agency has often been understood through discourses of rationality linked to the capacity to act based on logic and reasoning and the ability to make choices beyond biological needs or urges. Framing agency in terms of rationality is problematic both for persons with disabilities and animals. Critical theorists across the posthumanities have sought to expand our notion of agency to account for the reality that the way we navigate and shape the world exceeds rationality and intentionality: humans are driven by instincts and emotions and pheromones among many other factors, just as all animals are, and are continuously in emergent relations with the world around us (64, 65). All beings shape the world and influence events and environments, whether they do so intentionally or not (66).

Differing understandings of agency impact how we consider things like autonomy, choice, and control. For instance, Wiebe and Mullin [(56), p. 1] take a relational view that understands autonomy as “self-governance in the service of personally meaningful goals, values and commitments.” Similarly, S. Taylor [(67), p. 200] describes what she means by individuals with disabilities being in control of their life as follows: “independence is more about individuals being in control of their own services (be it education, plumbing, electrical, medical, dietary, or personal care) than it is about individuals being completely physically self-sufficient.” CDS scholars highlight that there are many ways in which individuals with disabilities or chronic illnesses express agency in the context of healthcare, from the work that is done to care for oneself, to seeking out information to guide healthcare decision-making (68, 69).

In the context of intellectual disability, conventions around informed consent have historically resulted in the perpetuation of exclusions (70). Those doing work with and speaking from the disability community emphasise that even where decision-making capacity and comprehension may be limited, or absent, it is imperative to take into account individuals’ preferences. As Noorlandt et al. [(71), p. 882] write, “People with [intellectual disabilities] have the right to be supported in making choices even if they cannot make such decisions by themselves.” In the context of EoL, there is still an obligation for healthcare providers and caregivers to find ways to include individuals in shared decision making about their preferences. There are many ways to express preferences when there are barriers to communication, for instance through: “behaviour, vocalization, vocal pitch, muscle tone, facial expression, eye movement, self-harm, breath” [(72), p. 1027]. The role of caregivers, family members, and healthcare professionals is to acknowledge, interpret, and respond to these communications and expressions of preference (72). Overall, very few studies have described processes of EoL decision-making in which persons with intellectual disabilities actively participated. Despite a lack of best practices, there are processes through which “decisions can be aligned to the values and preferences of a person with [intellectual disabilities]” [(71), p. 892].

Choice, autonomy, and control have also been key themes in the ‘right to die’ movement (73). Central to this is an understanding that “[t]he individual alone defines at which point her life has or will become meaningless or unbearable and when the time is ripe to die a good, still dignified death” [(74), p. 76]. It is a violation to impose upon another a judgement of when it is appropriate to die. Even in the case where autonomy might be compromised by oppressions and unjust social systems, there are still arguments that MAiD should be available, as negating this option perpetuates harms within unjust systems by further reducing individuals’ autonomy in decision-making around their own EoL (56).

#### 4.3.1 Relevance to veterinary ethics and euthanasia

There is still a tendency within veterinary ethics to consider all animals as lacking autonomy, meaning “they are not able to tell us their preferences and we are not able to explain future benefits from current treatments or actions to them” [(22), p. 3]. However, there can be a recognition of animals having preferences which can be expressed and interpreted, especially through nonverbal communication and by those who know the individual well. There are therefore questions about creating space wherein an animal “expresses their preference to continue living or rather to end their life” [(22), p. 3]. Similarly, the IAAHPC Guidelines (57) state that in EoL decision-making for companion animals, carers “should remain attuned to an animal’s ‘will to live’.”

There is also a tendency to foreground or support the agency and preferences of human caregivers over those of companion animals themselves (22, 75). For instance, in a study by Persson et al. (22), they discuss how sedation of a patient such that they will die naturally while asleep and not experience suffering is common in human medicine and positioned as providing a ‘dignified’ death. It is not common practice in veterinary medicine, compared with euthanasia, however a not inconsiderable number of veterinarians expressed that they would select such an option, and justified it as a means of giving the human family members more time to say goodbye. Thus (hypothetical) decision-making was based not on the choice, agency, or best interest of the animal patient, but rather out of consideration for the emotional wellbeing of their human caregiver(s).

Similarly, within the AVMA Guidelines on Euthanasia [(20), p. 8], there is note of the importance of the “autonomy of their clients to make decisions on behalf of their animals.” The IAAHPC Guidelines (57), however, provide more consideration for the agency of the animal patient. The Guidelines specify that:

*It is important to base decisions about care on an understanding of the animal’s feelings, experiences, and **preferences**. We can gather a great deal of information by carefully observing an animal’s behavior, physiological state, and **nonverbal communications** (Wemelsfelder 2007). Knowledge of species-specific behavior is extremely important, as is an attunement to **individual personality*** (p. 9, emphasis added).

They further state that “though it can be difficult to determine the animal’s own wishes, they must be considered” (p. 42). Schuurman (76) writes about ‘giving voice’ to the animal by ‘interpreting behaviour’—meaning responding to the animals’ physical, mental, and emotional displays. Morgan [(63), p. 46] agrees that “quasi-autonomy, expressed through individual patient preferences, should influence QoL assessments or predictions.” MacMartin et al. (77) begin to do this in their paper on veterinarian’s ‘*I know*’ responses to animals’ shows of distress during veterinary appointments. This response aligns to the embodied and vocal distress of the animals, recognising animals’ own embodied responses to procedures, while also claiming a shared understanding of the pain and discomfort being experienced. The *I know* claim attends to patient resistance but at times this is still overridden by the goal of the appointment. Overall, despite such sentiments, in a context where animals are considered property not persons under the law the emphasis has been on human client autonomy and choice in veterinary medicine, not on the animal patient’s, and most EoL decisions are made on behalf of animals in a more paternalistic fashion (63).

### 4.4 Care and power

The final theme is care and power, which have also been central to CDS and its consideration of EoL. Tronto and Fisher (78) define care as:

*A species activity that includes everything that we do to maintain, continue, and repair our ‘world’ so that we can live in it as well as possible. That world includes our bodies, ourselves and our environment, all of which we seek to interweave in a complex, life sustaining web* (p. 40).

Puig de la Bellacasa (79) builds on this definition to delineate three dimensions of care: work/labour, affect/affections, and ethics/politics. They emphasise that caring is a doing, involves emotions, and is always inherently political. Care also entails a network of actors and actions: it does not have to be solely from one actor to another, and those receiving care can also give care. Milligan and Wiles (80) outline this multidirectional nature of care as involving networks, not dyads, that are characterised by different kinds of care that can be extended through different types of reciprocity by multiple actors. These multiple dimensions highlight the problematic nature of the cared-for/caregiver dyad, as traditionally conceived.

Care as multidirectional challenges the often-understood narrative of care as a contractual relationship wherein the person with disabilities is dependent on another (able-bodied) person for the completion of important daily tasks. Care in this sense, as dependency, is situated as negative, stigmatised, and associated with burden, vulnerability, and reliance. Social attitudes towards ideas of dependence are often prevalent in hate crimes towards people with disabilities (81), framing dependence as entirely negative and targeting persons with disabilities as ‘spongers’ or ‘parasites’ (82). This dependency, once largely controlled by the state through spatial segregation through institutional incarceration, has been moved to the spaces of family and community. This is entangled with a long history of gendered and racialized practices of caregiving, where women and people of colour are often providing care for the elderly and persons with disabilities (83–86).

While dependency is framed as negative, independence is seen as a positive, and as the end goal for persons with disabilities. Such framings often ignore the structures and relations persons with disabilities rely on. E. Hall (87) describes how sites of paid employment and independent living, which are designed to be inclusionary, can still be sites of exclusion for people with learning disabilities, whereas spaces of assumed exclusion, such as care homes and unpaid work, can often help people feel more included. N. Watson et al. (88) position ‘(inter)dependence’ as more suitable in that it allows for the option of living and taking care of oneself by having assistance when and how one requires. It acknowledges more widely that everybody has certain dependencies within their own lives. In this sense the emphasis is on mutualism and creating social relations (89, 90), as “disability necessarily demands and affirms interdependent connections with other humans, technologies, non-human entities, communication streams and people and nonpeopled networks” [(91), p. 348].

Care is also inevitably embroiled with power. Within medicine, it structures the patient-physician relationship, especially in the case of individuals with disabilities, for instance in “assumptions that the science knowledge base of the physician trumps the personal and social knowledge base of the person with a disability” [(40), p. 122]. Price [(92), p. 5] emphasises that “intimate relationships are always emerging in the context of larger systems of power and violence… we cannot choose sides among independence, dependence, and interdependence, but rather must constantly navigate the tension among these concepts.” Care is thus inherently ambivalent - it can be beautiful, rewarding, genuine, oppressive, begrudging, violent, and anything in between. CDS scholars remind us that this ambivalence is not necessarily something to be resolved, but rather a reality that must simply remain acknowledged and explored, but will remain irresolvable (93).

#### 4.4.1 Relevance to veterinary ethics and euthanasia

The above discussion encourages a rethinking of how animals in need of care, and EoL care in particular, are positioned. Schuurman (76) discusses the complexity of the relationship between killing and caring inherent in euthanasia. Owners are seen to have an “ethical duty of care towards the pet,” which includes how the animal’s needs are supported at the end of life, and justifications for decision-making around euthanasia or other EoL processes [(76), p. 211]. Schuurman [(76), p. 208] writes that “the animal can be killed at the same time that its relationship with humans is celebrated – an act of responsible killing and of care, with a possibility to provide the animal a good ending to its life.” Similarly, Hurn and Badman-King [(62), p. 139] write that “paying mindful attention to the diverse ways in which individual animals are cared for as they die reveals the potential violence inherent in both palliative care leading to natural death, and euthanasia, blurring perceptions of good and bad death in both veterinary and human medicine.” One such example comes from Dickinson and Hoffmann (94) who discuss the role of the human companion in staying with the animal companion or leaving the room during veterinary euthanasia. For those that stayed they saw this as a moral obligation to their animal companion, for those that left, their reasons did not reflect a lack of care, but rather feelings of guilt and sadness. This blurs the perception of good and bad care during euthanasia from a human companions’ view. This can be experienced through different engagements with companion animals such as using touch during veterinary care and procedures (95). Llewellyn et al. (95) show how ‘coalitions of touch’ can produce meaningful engagements of care for animals, but also how animals are placed under, and resist, human constraints in veterinary procedures. The coalitions of care shown during appointments show both soothing animals through stroking and holding in a manner the animal likes, but also resistant through the use of a muzzle.

“Proper conduct” of care and killing involves both the practice of euthanasia and its context. What is interesting here is the idea of knowing *when* this care is needed. This links to anthropocentric power over animals as many will interpret an animal’s QoL or rely on others to do so. Dickinson et al. (96) talk about how caretakers of companion animals rely on veterinarians for their expertise regarding euthanasia, but in Redmalm’s (97) study many people interpreted the communication and bodily signs of their aging and ill animals as a reason for euthanasia. The decision of EoL care then is a human decision but animals’ own embodied capacities may shape this. The power over care, and in particular ‘good care’ is one in human hands.

In the human medical context, there is an emphasis that within the context of disability especially, QoL needs to be a subjective self-assessment, not externally interpreted. In the case of animals who cannot straightforwardly communicate their own perception of their QoL, there are power relations at play in interpreting this for another being. There is a risk of paternalism in making decisions on behalf of the other, when we assume we know what is best for them. The IAAHPC Guidelines, though robust in many ways, do use language that reflects this, including that: “witnessing and supporting an animal’s dying process can provide a sense of final gift-giving and good parenting” [(57), p. 29]. Although ‘pet parent’ discourses are common and companion animals are increasingly positioned as members of the family, discourses around ‘fur babies’ that position companion animals as children in heteronuclear family units have been critiqued by CAS scholars, who highlight the problematic nature of this infantilization (98, 99).

Overall, CDS helps us think about the ambivalence and power inherent in caring for companion animals, which is relevant in end-of-life contexts. Ashall (2) writes that:

*…there is something particularly difficult about a vet’s involvement in human-animal relationships which can often combine love, neglect, tenderness and violence. In order to understand this more fully we may choose to consider the uncomfortable possibility that these feelings matter, in an ethical sense.*

She concludes that there is a need for more engagement with feminist ethics of care within veterinary medicine and ethics. We agree that there is great potential to enrich veterinary ethics through further engagement with critical theories such as intersectional feminism and CDS.

## 5 Conclusion

Much of veterinary dialogue and practice around EoL and euthanasia neglects to consider the political and cultural dimensions of death and dying, which reduces the ability to address questions like: what future potential goods (positive affective states, meaning) could an animal experience by continuing to live; what constitutes a life worth living; and how can we make this decision for another being? These are questions that have been subject to extensive dialogue within CDS, and we argue that insights from CDS offer an opportunity to consider questions of death and dying, EoL practices, and the ethics of euthanasia with more nuance and complexity.

In particular, this paper considers four areas in which we feel veterinary ethics could be informed by dialogues in CDS. First, critiques of the *dis/ability binary* and associated hierarchies raise questions about how animal disability and illness are understood, and around challenging questions like psychological illness and behavioural euthanasia. Second, nuanced engagements with questions of *a life worth living* and QoL emphasise the importance of individual animal experiences, personality, and the foregrounding of ‘what is important for the animal’. Third, dialogues around *choice and agency* critique the tendency to focus on human owner choice alone, rather asking what opportunities there are to listen to the preferences of animals themselves. Finally, engaging with *care and power* highlight the ambivalent nature of caregiving, of euthanasia as a practice of care, and the power intrinsic to making EoL decisions on behalf of another.

The AVMA Guidelines on Euthanasia [(20), p. 7] write that: “What constitutes a good life and what counts as an impoverished life, or one that has limited quality such that the death of the animal is the most humane option, are research areas in need of further study by the veterinary and ethics communities.” We hope that such future explorations incorporate some of the complexity and nuance offered by CDS, along with other critical theories.

## Footnotes

^1^We use the term ‘owner’ as this is common language in the discipline, but do not support the reduction of animals to human property. Other terms such as ‘guardian’ could be seen as preferable, but also imply paternalism or unequal power relationship with companion animals.

^2^See scales used by Lynch et al. (17), Tatlock et al. (18), and Roberts et al. (100).

^3^See, for example: https://www.catster.com/lifestyle/disabled-cat-home-accessible; https://www.adventurecats.org/pawsome-reads/cats-with-disabilities-can-adventure-too/
